# MNCLCDA: predicting circRNA-drug sensitivity associations by using mixed neighbourhood information and contrastive learning

**DOI:** 10.1186/s12911-023-02384-0

**Published:** 2023-12-18

**Authors:** Guanghui Li, Feifan Zeng, Jiawei Luo, Cheng Liang, Qiu Xiao

**Affiliations:** 1 School of Information Engineering, East China Jiaotong University, Nanchang, China; 2 College of Computer Science and Electronic Engineering, Hunan University, Changsha, China; 3 School of Information Science and Engineering, Shandong Normal University, Jinan, China; 4 College of Information Science and Engineering, Hunan Normal University, Changsha, China

**Keywords:** circRNA-drug sensitivity associations, Random walk with restart, Contrastive learning, Graph convolutional network

## Abstract

**Background:**

circRNAs play an important role in drug resistance and cancer development. Recently, many studies have shown that the expressions of circRNAs in human cells can affect the sensitivity of cells to therapeutic drugs, thus significantly influencing the therapeutic effects of these drugs. Traditional biomedical experiments required to verify this sensitivity relationship are not only time-consuming but also expensive. Hence, the development of an efficient computational approach that can accurately predict the novel associations between drug sensitivities and circRNAs is a crucial and pressing need.

**Methods:**

In this research, we present a novel computational framework called MNCLCDA, which aims to predict the potential associations between drug sensitivities and circRNAs to assist with medical research. First, MNCLCDA quantifies the similarity between the given drug and circRNA using drug structure information, circRNA gene sequence information, and GIP kernel information. Due to the existence of noise in similarity information, we employ a preprocessing approach based on random walk with restart for similarity networks to efficiently capture the useful features of circRNAs and drugs. Second, we use a mixed neighbourhood graph convolutional network to obtain the neighbourhood information of nodes. Then, a graph-based contrastive learning method is used to enhance the robustness of the model, and finally, a double Laplace-regularized least-squares method is used to predict potential circRNA-drug associations through the kernel matrices in the circRNA and drug spaces.

**Results:**

Numerous experimental results show that MNCLCDA outperforms six other advanced methods. In addition, the excellent performance of our proposed model in case studies illustrates that MNCLCDA also has the ability to predict the associations between drug sensitivity and circRNA in practical situations.

**Conclusions:**

After a large number of experiments, it is illustrated that MNCLCDA is an efficient tool for predicting the potential associations between drug sensitivities and circRNAs, thereby can provide some guidance for clinical trials.

## Background

Circular RNAs (circRNAs) are novel noncoding RNA molecules with continuous circular structures that belong to the noncoding cancer genome family [1, 2]. circRNAs include four categories: intergenic circRNAs [3], exon–intron circRNAs [4], intronic circRNAs [5], and exonic circRNAs [6]. In recent years, with the development of high-throughput sequencing technology, it has been found that circRNAs can be implicated in many important biological processes [7]. For example, circRNAs, as the "sponges" of miRNAs or competitive endogenous RNAs, competitively bind to miRNAs to influence the regulation of target genes by miRNAs [8]. A single circRNA has the ability to bind to multiple units of RNA-binding proteins, thus serving as a reservoir for these RNA-binding proteins [9]. At present, much evidence [10, 11] suggests that some circRNAs are translated into proteins through the rolling circle amplification mechanism [12]. These experimentally verified biological functions indicate that circRNAs can become a class of potential medical diagnostic markers in clinical settings.

Recent research has shown that the expressions of circRNAs can have significant impacts on cellular drug sensitivity. For example, Joseph et al. [13] found that CircCCDC66 was highly expressed in lung adenocarcinoma cells, thereby reducing the sensitivity of the cells to cisplatin. Jin et al. [14] found that CircPAN3 increased in the acute myeloid leukaemia (AML) cells and bone marrow cells of relapsed patients, while some target miRNAs decreased. Without affecting the apoptosis of basal cells, the knockout of CircPAN3 restored the sensitivity of the AML cells to chemotherapy drugs. Zhang et al. [15] found that a circRNA (Hsa_circ_0005379) was downregulated in oral cancer tissues, while its elevation reduced cell proliferation, induced apoptosis, and increased the sensitivity of cancer cells to cetuximab. It is crucial to identify the relationships between circRNAs and drug sensitivities, which have value for both disease treatment and drug discovery. To explore the influences of circRNAs expression on drug sensitivities, Ruan et al. [16] utilized several circRNA recognition methods to judge the circRNA expressions in approximately 1000 human cancer cell lines, and they discovered that the expressions of these circRNAs are significantly related to some drug responses. It is worth noting that thus far, our understanding of the relationships between drug sensitivities and circRNAs is still incomplete.

Verifying the relationships between drug sensitivities and circRNAs through traditional biomedical experiments can be both costly and time-consuming. Therefore, developing an effective and precise computational approach for predicting the associations of circRNAs with drug sensitivities could significantly reduce the cost of biomedical experiments. As a pioneer study, Deng et al. [17] first presented a computational deep learning model called GATECDA for excavating the associations between drug sensitivities and circRNAs, which uses a graph attention autoencoder (GATE) to learn low-dimensional representations from drug and circRNA networks and finally uses deep neural networks for classification to predict the novel associations between drug sensitivities and circRNAs. Subsequently, Chen et al. [18] developed a new model called MNGACDA, which utilizes multiple types of data from drugs and circRNAs to create a multimodal network. Then, a node-level attention graph autoencoder is used to extract the low-dimensional embeddings of drugs and circRNAs from the multimodal network. Finally, using the circRNA and drug embeddings, an inner product decoder is used to infer the potential associations between the drug sensitivities and circRNAs. Comprehensive experiments performed on the above two computational methods demonstrate that correlation-based computational methods are effective in terms of predicting the associations between drug sensitivities and circRNAs. As described in the aforementioned research, there are still very few computational methods in this area. To the best of our knowledge, only GATECDA and MNGACDA are currently used for predicting the associations between circular RNAs and drug sensitivities. It is important to note that the known circRNA-drug sensitivity associations validated through biomedical experiments are incomplete, and many associations remain undiscovered. Consequently, the development of more accurate computational methods is necessary to make more reliable predictions regarding the sensitivity associations between circRNAs and drugs, thus improving the efficiency of developing related drugs.

In this paper, we advance a new computational framework, called MNCLCDA, for predicting the potential associations of circRNAs with drug sensitivities. First, we use host gene sequence information, drug structure information and GIP kernel information to obtain the comprehensive similarity between circRNAs and drugs. Due to the existence of noise in similarity information, we design a preprocessing method based on random walks with restarts for the circRNA and drug similarity networks to efficiently capture the useful features of circRNAs and drugs, respectively. Then, we use mixed-neighbourhood graph convolution on the circRNA-drug sensitivity bipartite network to obtain node embeddings. At the same time, we design a contrastive learning task to make the encoder more discriminative and enhance the robustness of our model. Finally, the double-Laplacian graph-regularized least-squares method is used to infer potential associations between drug sensitivities and circRNAs through the kernel matrices in the circRNA and drug spaces. To assess the performance of MNCLCDA, we perform cross-validation experiments using a benchmark dataset and compare it with six relevant state-of-the-art methods. Our experimental results indicate that MNCLCDA performs better than the existing related methods. Furthermore, we conduct an ablation study on the model. Finally, we perform a case study involving four drugs, which shows that MNCLCDA can effectively screen for circRNAs that are related to drug sensitivities. Therefore, the above experimental results indicate that MNCLCDA can infer the sensitivity associations between circRNAs and drugs, thereby providing guidance for clinical trials.

## Methods

### circRNA-drug sensitivity associations

We download the circRNA-drug sensitivity association dataset from reference [17], where Deng et al. [17] collected and organized the association data between circRNA and drug sensitivity from the circRic database [16]. Here, the drug sensitivity and circRNA data come from the GDSC database [19], which provides 80,076 circRNA-drug sensitivity association data consisting of 250 drugs and 404 circRNAs. After using the Wilcoxon test to identify the relationship between each circRNA and drug sensitivity pair, correlations with false discovery rates < 0.05 are defined as significant associations. In our research method, we use these significant associations as our benchmark dataset of circRNAs and drug sensitivities, including a total of 4134 associations, 218 drugs and 271 circRNAs. On the basis these significant associations, we finally construct the association matrix $Y\in {R}^{271\times 218}$ between the circRNAs and drugs. In the association matrix $Y$, rows represent circRNAs, and columns represent drugs. If an element *Y* (*i, j*) = 1, it means that the corresponding drug and the circRNA are sensitive; otherwise, *Y* (*i, j*) = 0. Therefore, we can use the circRNA-drug sensitivity associations to construct a bipartite network *A*, and we define the adjacency matrix $A\in {\mathbb{R}}^{(nc+nd)\times (nc+nd)}$ of the bipartite network as follows:

1 $$A=\left[\begin{array}{cc}0& Y\\ {Y}^{T}& 0\end{array}\right]\in {{\varvec{R}}}^{\left(Nc+Nd\right)\times \left(Nc+Nd\right)}$$

Furthermore, we access the host gene sequence data of circRNAs from the NCBI Gene Database [20] and obtain the structural information of drugs from the PubChem database [21]. We subsequently compute their respective similarities using appropriate methods.

### Sequence similarity between the host genes of circRNAs

We use the circRNA host gene sequence information to calculate the similarity between the circRNAs. The similarity between two circRNA fragments is measured using the Levenshtein distance measure [22], which is a tool for calculating the difference between two strings. We denote the similarity between circRNAs by $SC\in {R}^{271\times 271}$. The procedure of circRNA sequence similarity determination is calculated as follows:

2 $${SC}_{\text{leven }}\left({c}_{i},{c}_{j}\right)=1-\frac{\text{ trans }}{len\left({c}_{i}\right)+len\left({c}_{j}\right)}$$

where ${\text{trans}}$ represents the lowest cost of conversion between circRNAs and $len\left(\bullet \right)$ represents the size of the circRNA sequence.

### Structural similarity of drugs

The functions of drugs are largely determined by their chemical structures; therefore, we can obtain the similarity between drugs by comparing their chemical structures. After obtaining the chemical structure information of the drugs from the PubChem database, we first utilize the RDKit [23] to compute the topological fingerprint of each drug, and then compute their structural similarities using the Tanimoto method [24]. Therefore, we can obtain a structural similarity matrix between drugs, which is expressed as $SD\in {R}^{218\times 218}$.

### Gaussian interaction profile kernel similarity

To discover additional useful similarity data, we apply the Gaussian interaction profile kernel function to compute the Gaussian kernel similarities between drugs and circRNAs. The GIP kernel similarity measure is extensively employed to calculate similarity in the field of bioinformatic association prediction [25–27]. Therefore, we can calculate the GIP kernel similarities of circRNAs using the following equation:

3 $$GC\left(i,j\right)=exp\left({-r_c\parallel Y\left(i,:\right)-Y\left(i,:\right)\parallel}^2\right)$$

4 $${r}_{c}=1/\left(\frac{1}{{n}_{c}}\sum_{i=1}^{{n}_{c}}\| Y(i,:){\| }^{2}\right)$$

where *Y* (*i*,:) represents the *i*-th row of the association matrix *Y*. The parameter ${r}_{c}$ represents the bandwidth, while ${n}_{c}$ represents the total count of circRNAs. Similarly, the GIP kernel similarity between drug *i* and drug *j* can be computed using the following equation:

5 $$GD\;\left(i,j\right)=exp\left({-r_d\parallel Y\left(:,i\right)-Y\left(:,j\right)\parallel}^2\right)$$

6 $${r}_{d}=1/\left(\frac{1}{{n}_{d}}{\sum }_{i=1}^{{n}_{d}}{\Vert Y(:,i)\Vert }^{2}\right)$$

where *Y* (: *i*) represents the *i*-th column in the association matrix *Y*, and ${r}_{d}$ is similar to ${r}_{c}$.

### Integrated similarity for circRNAs and drugs

Above, we calculate two similarity matrices for circRNAs and drugs. To supplement the biological information and improve the similarity between the drugs and circRNAs, we construct a comprehensive circRNA similarity matrix by integrating the circRNA sequence and Gaussian kernel similarities. If sequence similarity is observed between two circRNAs, the comprehensive similarity of the circRNAs is defined as the average of the sequence similarity and Gaussian kernel similarity; otherwise, it is the Gaussian kernel similarity. We define the comprehensive similarity *CS* between circRNAs as follows:

7 $$C{S}_{ij}=\left\{\begin{array}{c}\frac{\left(S{C}_{ij}+G{C}_{ij}\right)}{2}\hspace{1em},ifSC\left(i,j\right)\ne 0\\ G{C}_{ij}\hspace{1em}\hspace{1em}\hspace{1em},otherwise\end{array}\right.$$

Similarly, the comprehensive similarity matrix of the drugs can be computed as follows:

8 $$D{S}_{ij}=\left\{\begin{array}{c}\frac{\left(S{D}_{ij}+G{D}_{ij}\right)}{2}\hspace{1em},ifSD\left(i,j\right)\ne 0\\ G{D}_{ij}\hspace{1em}\hspace{1em}\hspace{1em},otherwise\end{array}\right.$$

### MNCLCDA algorithm

**Fig. 1:**
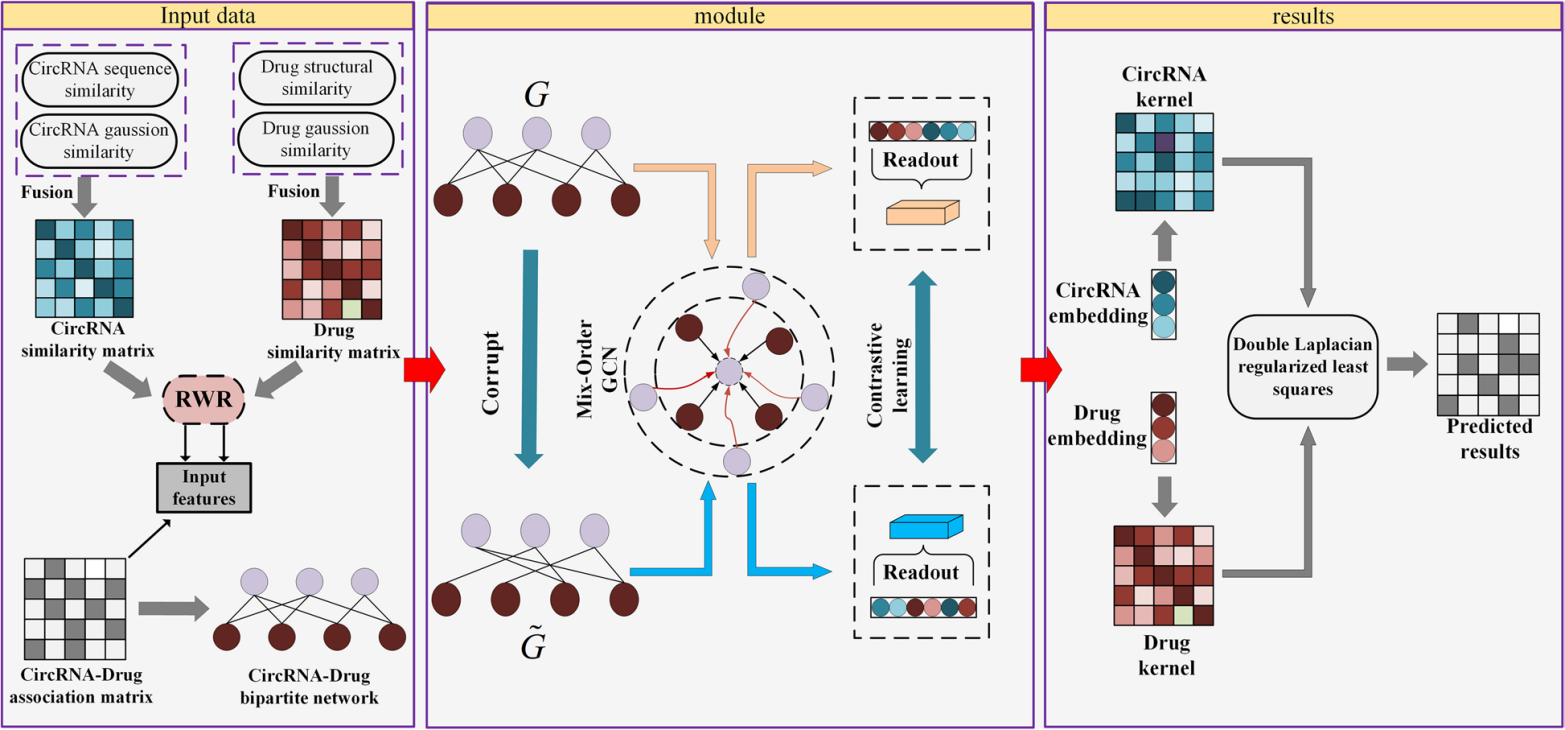
Flowchart of the MNCLCDA model

In this work, we propose a model called MNCLCDA to explore the relationships between circRNAs and drug sensitivities. As shown in Fig. 1, MNCLCDA mainly consists of the following steps. First, in the data preprocessing part, we use the sensitivity associations between drugs and circRNAs to construct a bipartite network and then use the comprehensive similarity data of the circRNAs and drugs as RWR inputs to learn potential features from the information possessed by the low-order and high-order neighbours. Next, we combine the above features and association matrix to generate new feature representations for the circRNAs and drugs. In the second step, we use a mixed neighbourhood graph convolution to learn the potential embeddings of the circRNAs and drugs. Third, we separately compute the Gaussian kernel similarity based on the circRNA and drug embeddings. Fourth, we predict the potential associations of the circRNAs and drugs using the double Laplacian-regularized least-squares method in the circRNA and drug kernel spaces. Finally, we use contrastive learning as an auxiliary task to make the model more discriminative and enhance its robustness.

### Feature processing for circRNAs and drugs

As previously described, *DS* and *CS* are comprehensive similarity matrices for drugs and circRNAs, respectively. In the comprehensive similarity matrix, each row or column represents the similarity distribution of circRNAs (or drugs), which can be regarded as a feature vector for these circRNAs (or drugs). However, the calculated similarity matrix may generate some noise due to the presence of false positives or the limitations of the calculation approach. To decrease the effect of this noise, we use a random walk with restarts (RWR)-based method to obtain features from the similarity matrix. RWR is a network embedding algorithm that extracts the similarity between nodes through random walks; it can capture not only local information but also the global information of the network. In previous studies, random walks were often used to denoise images [28] and preserve neighbour information in feature engineering tasks [29], and they have also been widely applied in the field of bioinformatics [30, 31]. Therefore, we apply RWR to our problem as well. The RWR calculation method [32] is defined as follows:

9 $${\overrightarrow{r}}_{l}=c\widetilde{W}{\overrightarrow{r}}_{l}+\left(1-c\right){\overrightarrow{e}}_{l}$$

where $\widetilde{W}$ denotes the transition probability matrix obtained after normalization and ${\overrightarrow{e}}_{l}$ denotes the row vector of the similarity matrix. *c* is the probability of restarting. In the following experiments, we set *c* = 0.3, and ${\overrightarrow{r}}_{l}$ is the score vector obtained after multiple rounds of RWR computations.

After separately performing RWR on the drug and circRNA similarity matrices, we obtain a probability distribution vector for each drug and circRNA. Therefore, we recombine the probability distribution vectors into a new drug feature matrix ${F}_{d}\in {{\varvec{R}}}^{Nd\times Nd}$ and a new circRNA feature matrix ${F}_{c}\in {{\varvec{R}}}^{Nc\times Nc}$. Finally, we combine the circRNA-drug sensitivity association matrix *Y* with ${F}_{d}$ and ${F}_{c}$ to form the initial feature matrix $X\in {{\varvec{R}}}^{(Nc+Nd)\times (Nc+Nd)}$ of the model, which is described as follows:

10 $$X=\left[\begin{array}{cc}{F}_{c}& Y\\ {Y}^{T}& {F}_{d}\end{array}\right]\in {{\varvec{R}}}^{\left(Nc+Nd\right)\times \left(Nc+Nd\right)}$$

### Mixed-neighbourhood graph convolutional network

A graph convolutional network (GCN) [33] is a kind of deep learning model that can extract low-dimensional representations and is applicable to graph structures. A GCN is cleverly designed to extract features from graphs so that we can obtain graph embeddings to solve downstream problems such as graph classification, link prediction, and node classification. GCNs are extensively employed in bioinformatics field [34–36]. In a normal GCN, each node representation is updated by aggregating the information acquired from its direct neighbours because the network only pays attention to the first-order neighbourhood information of the nodes every time without considering other order information, which makes it poor at capturing higher-order domain information and can easily cause node oversmoothing. Many studies have shown that fusing mixed neighbourhood information from neighbours can enable GCNs to learn better node representations, which can help improve the resulting predictions for downstream tasks [37, 38]. Therefore, we design a mixed-neighbourhood graph convolutional network that aggregates information not only from direct neighbours but also from multiorder neighbours directly. In the constructed bipartite network, the direct neighbours of each node are heterogeneous nodes, and its second-order neighbour nodes are homogeneous nodes. The mixed-neighbourhood graph convolutional network can gather information about circRNAs and drugs simultaneously, and the use of multiple mixed-neighbourhood graph convolutional networks can also broaden the information obtained by nodes and reduce oversmoothing. Specifically, we construct adjacency matrices with different orders based on the bipartite network, then use the GCN for feature extraction, and finally concatenate these features. The computation process is as follows:

11 $${\overline{A} }_{ij}^{n}=\left\{\begin{array}{ccc}\pi 1& ,\text{ if} \, {A}_{ij}^{n}\ne 0& \\ 0& , \, {\text{otherwise}}& ,n\in \{\mathrm{1,2},\dots ,N\}\end{array}\right.$$

12 $${H}^{\left(l+1\right)}={\parallel }_{i\in n}\sigma \left({\widetilde{A}}^{i}{H}^{l}{W}_{i}^{l}\right)$$

where ${\widetilde{A}}^{i}={\widetilde{D}}_{i}^{-\frac{1}{2}}\left({\overline{A} }^{i}+I\right){\widetilde{D}}_{i}^{-\frac{1}{2}}$ is a symmetric normalized adjacency matrix with self-connection, ${H}^{\left(0\right)}=X$, $I$ is the identity matrix with the same shape as ${\overline{A} }^{i}$, and ${\widetilde{D}}_{i}$ is the degree matrix of ${\overline{A} }^{i}+I$, which is also a diagonal matrix. $\sigma$ is the *ReLU* nonlinear activation function, and ${W}_{i}^{l}$ is a trainable matrix.

### Kernel matrices of graph embeddings

After applying the mixed-neighbourhood graph convolutional network, we obtain the final embeddings *H* of the nodes, which contain information from the mixed neighbourhood. We use the final embeddings *H* as feature vectors to compute the kernel matrices. We can divide the obtained embeddings $$H=\left[\begin{array}{c}{H}^{c}\\ {H}^{d}\end{array}\right]\in {\mathbf{R}}^{\left({N}_{c}+{N}_{d}\right)\times k}$$ into two parts, where ${H}^{c}\in {\mathbf{R}}^{Nc\times k}$ denotes the embeddings belonging to circRNAs and ${H}^{d}\in {\mathbf{R}}^{Nd\times k}$ denotes the embeddings belonging to drugs. We separately compute the Gaussian kernel matrices for the circRNA and drug embeddings by using GIP. The computational procedure is as follows:

13 $${\mathbf{K}}_{\mathbf{c}}=\mathrm{exp}\left(-{\gamma }_{h}{\parallel {H}^{c}\left(i\right)-{H}^{c}\left(j\right)\parallel }^{2}\right)$$

14 $${\mathbf{K}}_{\mathbf{d}}=\mathrm{exp}\left(-{\gamma }_{h}{\parallel {H}^{d}\left(i\right)-{H}^{d}\left(j\right)\parallel }^{2}\right)$$

where ${H}^{c}(i)$ and ${H}^{d}\left(i\right)$ represent the contours of row *i* in the circRNA and drug embeddings, respectively, and ${\gamma }_{h}$ represents the bandwidth of the Gaussian kernel.

### Double Laplacian-regularized least-squares method for prediction

We utilize the double Laplacian-regularized least-squares method [39] to infer the potential associations between drugs and circRNAs through the kernel matrices of the drugs and circRNAs. The loss function is defined as follows:

15 $$\begin{array}{c}{\mathcal{L}}_{1 }={\parallel {\mathbf{K}}_{c}{\mathrm{W}}_{c}+{\left({\mathbf{K}}_{d}{\mathrm{W}}_{d}\right)}^{\mathrm{T}}-2{\mathrm{Y}}_{\text{train }}\parallel }_{F}^{2}\\ +{\lambda }_{c}tr\left({\mathrm{W}}_{c}^{\mathrm{T}}{\mathbf{L}}_{c}{\mathrm{W}}_{c}\right)+{\lambda }_{d}tr\left({\mathrm{W}}_{d}^{\mathrm{T}}{\mathbf{L}}_{d}{\mathrm{W}}_{d}\right)\end{array}$$

where ||·||F is the Frobenius norm, ${Y}_{\text{train }}\in {\mathbf{R}}^{{N}_{c}\times {N}_{d}}$ is the adjacency matrix of the circRNA-drug sensitivity associations in the training set, ${W}_{c}$ and ${W}_{d}^{T}\in {\mathbf{R}}^{{N}_{c}\times {N}_{d}}$ are trainable matrices, ${K}_{c}\in {\mathbf{R}}^{Nc\times Nc}$ and ${K}_{d}\in {\mathbf{R}}^{Nd\times Nd}$ are the kernel matrices of the embeddings in the two feature spaces and the parameters ${\lambda }_{c}$ and ${\lambda }_{d}$ are the coefficients of the regularization terms. ${L}_{c}\in {\mathbf{R}}^{{N}_{c}\times {N}_{c}}$ and ${L}_{d}\in {\mathbf{R}}^{{N}_{d}\times {N}_{d}}$ are Laplacian regularization matrices defined as follows:

16 $${\mathbf{L}}_{c}={\mathbf{D}}_{c}^{-1/2}{\Delta }_{c}{\mathbf{D}}_{c}^{1/2} ,{\Delta }_{c}={\mathbf{D}}_{c}-{\mathbf{K}}_{c}$$

17 $${\mathbf{L}}_{d}={\mathbf{D}}_{d}^{-1/2}{\Delta }_{d}{\mathbf{D}}_{d}^{1/2},{\Delta }_{d}={\mathbf{D}}_{d}-{\mathbf{K}}_{d}$$

where ${\mathbf{D}}_{c}\left(k,k\right)=\sum_{t=1}^{Nc}{\mathbf{K}}_{c}\left(k,t\right)$ and ${\mathbf{D}}_{d}\left(k,k\right)=\sum_{t=1}^{Nd}{\mathbf{K}}_{d}\left(k,t\right)$ are diagonal matrices.

The final circRNA-drug sensitivity associations derived from the two feature spaces are combined as follows:

18 $${A}^{*}=\frac{{\mathbf{K}}_{c}{W}_{c}+{\left({\mathbf{K}}_{d}{W}_{d}\right)}^{T}}{2}$$

### Contrastive learning module

In recent years, contrastive learning has become a successful method for unsupervised representation learning and has also been successfully applied in the field of bioinformatics [40, 41]. We design a contrastive learning task inspired by deep graph Infomax (DGI) [42], which performs contrastive learning on the original bipartite graph $G$ and the corrupted graph $\widetilde{G}$ by maximizing the mutual information to enhance the robustness of the model. The process of the contrastive learning task can be outlined as follows.

First, we randomly shuffle the feature matrix $X$ after feature extraction to obtain the perturbed feature matrix $\widetilde{X}$. For the adjacency matrix of the bipartite graph is kept unchanged, and thus we construct a corrupted graph $\widetilde{G}=(A,\widetilde{X})$. DGI is capable of optimizing the learned embeddings from graph $G$ by maximizing the difference between the embeddings learned from the original graph $G$ and the embeddings learned from the corrupted graph $\widetilde{G}$. The essential purpose of this approach is to train a GNN encoder so that our prediction model learns node representations in a more discriminative manner. We encode the corrupted graph $\widetilde{G}$ by using the same GCN encoder as that used for the original graph $G$ and then obtain the embedding $\widetilde{H}\in {\mathbf{R}}^{\left({N}_{c}+{N}_{d}\right)\times k}$ of the corrupted graph from the damaged graph $\widetilde{G}$. Our goal for the contrastive learning task is as follows:

19 $${\mathcal{L}}_{2}=-\frac{1}{2\left|\mathcal{V}\right|}\left(\sum_{v\in \mathcal{V}}\mathrm{log\Gamma }(\overrightarrow{H},\mathrm{s})+\sum_{v\in \mathcal{V}}\mathrm{log}(1-\Gamma (\widetilde{\overrightarrow{H}},\mathrm{s}))\right)$$

where $\mathcal{V}$ represents the number of nodes in the graph,$\mathcal{V}=\left({N}_{c}+{N}_{d}\right)$, $s=R\left(H\right)=\sigma \left(\frac{1}{v}{\sum }_{i=1}^{v}{\overrightarrow{H}}_{i}\right)$, which is the graph-level embedding obtained through the readout function$R$, *R*: ${H\in \mathbf{R}}^{\left({N}_{c}+{N}_{d}\right)\times k}$→${s\in \mathbf{R}}^{1\times k}$, and $\Gamma (\overrightarrow{H},\mathrm{s})$ is a contrastive evaluator composed of bilinear functions$\sigma ({\overrightarrow{H}}^{T}\mathrm{Ws})$, which is used to evaluate the node-level embeddings and the graph similarity between the embeddings. Here, *W* is a trainable matrix, and $\sigma$ is the sigmoid nonlinear activation function.

Furthermore, we also extend the contrastive learning task from another perspective: by maximizing the difference $\widetilde{\mathbf{s}}=R(\widetilde{H})$ between the original graph node-level embedding *H* and the corrupted graph-level embedding, the contrastive loss function is as follows:

20 $${\mathcal{L}}_{3}=-\frac{1}{2\left|\mathcal{V}\right|}\left(\sum_{v\in \mathcal{V}}\mathrm{log\Gamma }(\widetilde{\overrightarrow{H}},\widetilde{\mathrm{s}})+\sum_{v\in \mathcal{V}}\mathrm{log}(1-\Gamma (\overrightarrow{H},\widetilde{\mathrm{s}}))\right)$$

### Optimization

To simultaneously perform the prediction and contrastive learning tasks, we optimize the objective loss function below, and the final loss function is represented as follows:

21 $$\mathcal{L}={\mathcal{L}}_{1 }+\alpha {\mathcal{L}}_{2 }+\beta {\mathcal{L}}_{3}$$

where the parameters α and β are used to balance the contributions of various tasks.

During the training process, we compute the partial derivatives of the parameters in the double Laplace-regularized least-squares method to directly obtain the iterative function, while the other parameters are optimized by Adam [43]. When optimizing the parameter ${W}_{c}$, we regard the parameter ${W}_{d}$ as a constant and compute the partial derivative of the loss function with respect to ${W}_{c}$ as follows:

22 $$\begin{array}{c}\frac{\partial J}{{W}_{c}}=2{\mathbf{K}}_{c}\left({\mathbf{K}}_{c}{\mathrm{W}}_{c}+{\mathrm{W}}_{d}^{\mathrm{T}}{\mathbf{K}}_{d}^{\mathrm{T}}-2{Y}_{\text{train }}^{\mathrm{T}}\right)\\ \end{array}+2{\lambda }_{C}{\mathbf{L}}_{C}{\mathrm{W}}_{c}$$

By letting $\frac{\partial J}{{W}_{c}}$= 0, we can obtain:

23 $$\begin{array}{c}\left({\mathbf{K}}_{C}{\mathbf{K}}_{C}+{\lambda }_{C}{\mathbf{L}}_{C}\right){\mathrm{W}}_{C}={\mathbf{K}}_{C}\left[2{Y}_{\text{train }}^{\mathrm{T}}-{\mathrm{W}}_{d}^{\mathrm{T}}{\mathbf{K}}_{d}^{\mathrm{T}}\right]\\ {\mathrm{W}}_{C}={\left({\mathbf{K}}_{C}{\mathbf{K}}_{C}+{\lambda }_{C}{\mathbf{L}}_{C}\right)}^{-1}{\mathbf{K}}_{C}\left[2{Y}_{\text{train }}^{\mathrm{T}}-{{\mathrm{W}}_{d}^{\mathrm{T}}\mathbf{K}}_{d}^{\mathrm{T}}\right]\end{array}$$

Similarly, the partial derivative of ${W}_{d}$ is calculated as follows:

24 $$\begin{array}{c}\frac{\partial J}{{W}_{d}}=2{\mathbf{K}}_{d}\left({\mathbf{K}}_{d}{\mathrm{W}}_{d}+{\mathrm{W}}_{c}^{\mathrm{T}}{\mathbf{K}}_{c}^{\mathrm{T}}-2{Y}_{\text{train }}^{\mathrm{T}}\right)\\ \end{array}+2{\lambda }_{d}{\mathbf{L}}_{d}{\mathrm{W}}_{d}$$

By letting $\frac{\partial J}{{W}_{d}}$= 0, we can obtain:

25 $$\begin{array}{c}\left({\mathbf{K}}_{d}{\mathbf{K}}_{d}+{\lambda }_{d}{\mathbf{L}}_{d}\right){\mathrm{W}}_{d}={\mathbf{K}}_{d}\left[2{Y}_{\text{train }}^{\mathrm{T}}-{\mathrm{W}}_{c}^{\mathrm{T}}{\mathbf{K}}_{c}^{\mathrm{T}}\right]\\ {\mathrm{W}}_{d}={\left({\mathbf{K}}_{d}{\mathbf{K}}_{d}+{\lambda }_{d}{\mathbf{L}}_{d}\right)}^{-1}{\mathbf{K}}_{d}\left[2{Y}_{\text{train }}^{\mathrm{T}}-{{\mathrm{W}}_{c}^{\mathrm{T}}\mathbf{K}}_{c}^{\mathrm{T}}\right]\end{array}$$

The pseudocode of MNCLCDA is shown in Algorithm 1:

**Figure:**
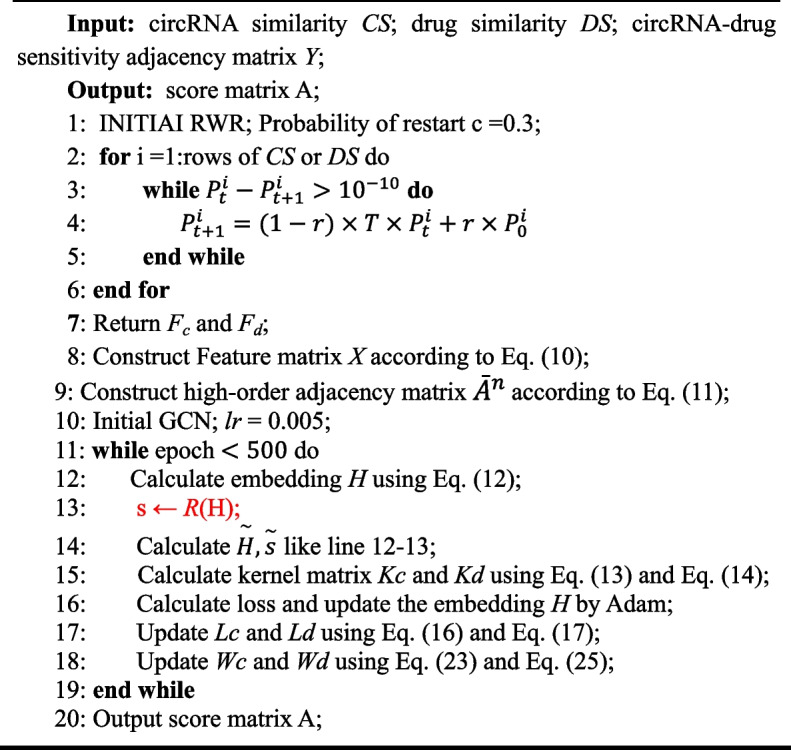
**Algorithm 1.** MNCLCDA Algorithm

## Results

### Evaluation metrics

Cross-validation is a typical method for evaluating the accuracy of a model. To fully assess the predictive performance of MNCLCDA, we perform fivefold and tenfold cross-validation experiments on circRNA and drug sensitivity datasets. Taking 5-CV as an example, we randomly select the same number of negative and positive samples and then split these samples into five identical sets. We sequentially use one of these five sets as the test set, and the other sets are used as the training set across five iterations to obtain accurate results. Similar to the 5-CV experiment, in the 10-CV experiment, we divide the samples into 10 subsets, one for testing and nine for training.

**Fig. 2:**
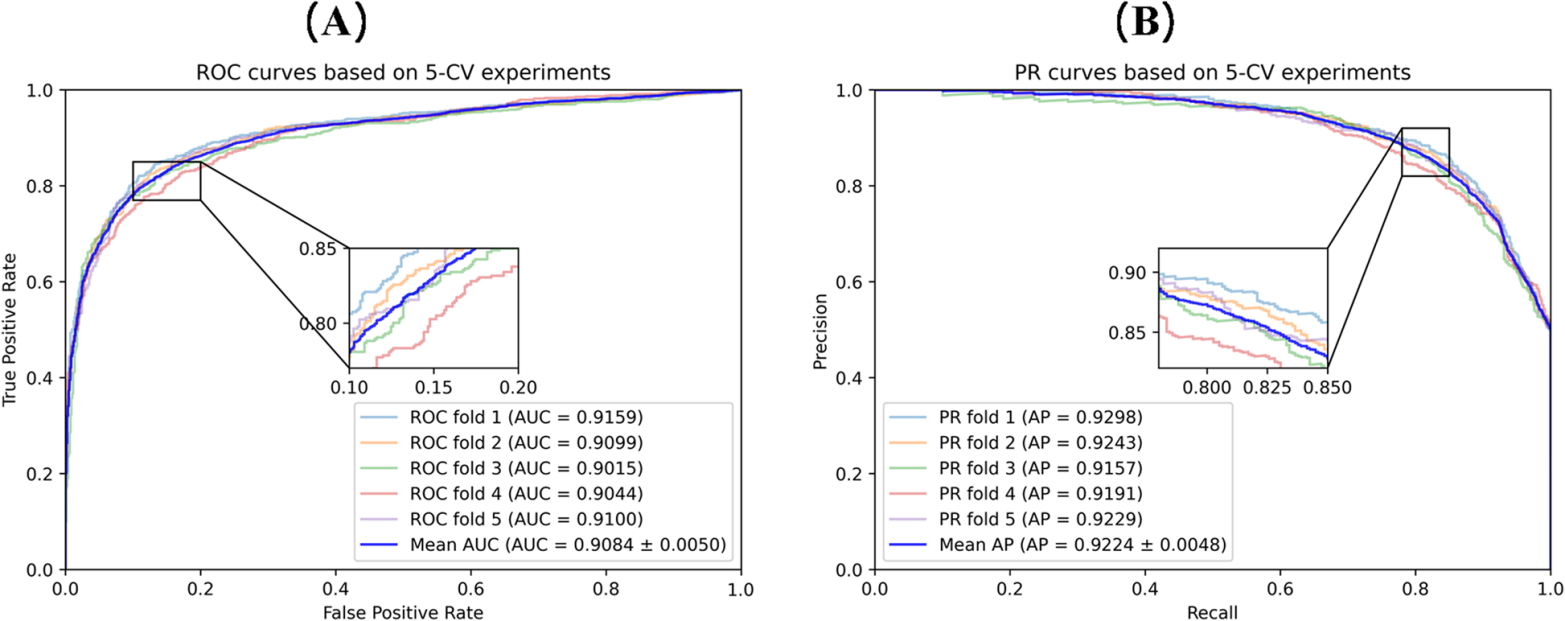
ROC curves and PR curves in fivefold cross validation

**Fig. 3:**
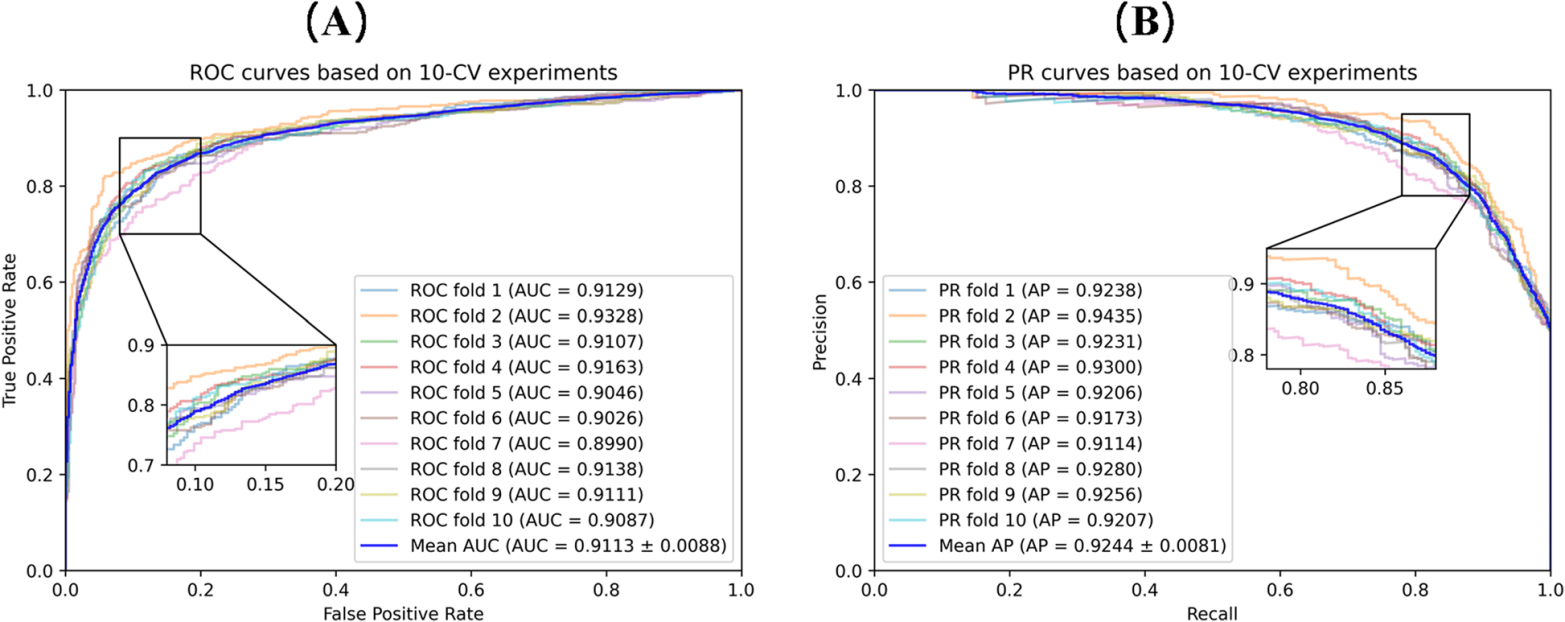
ROC curves and PR curves in tenfold cross validation

In the cross-validation experiments, we employ seven commonly used evaluation metrics to evaluate the predictive performance of MNCLCDA: the area under the precision-recall curve (AUPR), the area under the ROC curve (AUC), accuracy, recall, precision, specificity and the F1 score. These evaluation metrics are defined by Eqs. (26–30). In the equations, TP and TN represent the numbers of correctly identified unassociated and associated circRNA-drug pairs, respectively; FP and FN refer to the numbers of misidentified associated and unassociated circRNA-drug pairs, respectively. In addition, we plot receiver operating characteristic (ROC) curves and precision-recall (P-R) curves to visually display the performance of our model. The larger the AUC and AUPR value are, the better the predictive performance of the model. The ROC curve for the 5-CV case is shown in Fig. 2. The mean AUC of MNCLCDA is 0.9084, and the other metrics are shown in Table 1. The correlation averages of the AUPR, ACC, F1 score, precision, recall and specificity metrics are 0.9224, 0.8465, 0.8455, 0.8510, 0.8401 and 0.8523, respectively. The ROC curve for the 10-CV case is shown in Fig. 3, with an average AUC of 0.9113, and the other metrics are shown in Table 2.

**Table 1:** Comparison with other methods base on 5-CV

Method	F1-score	Accuracy	Recall	Specificity	Precision
MNCLCDA	**0.8455**	**0.8465**	0.8401	**0.8523**	**0.8510**
GATECDA	0.8237	0.8196	0.8361	0.8037	0.8125
MNGACDA	0.8416	0.8389	**0.8537**	0.8286	0.8308
VGAELDA	0.8197	0.8075	0.8278	0.8015	0.8123
MKGCN	0.8023	0.7985	0.8186	0.7889	0.7857
LAGCN	0.8084	0.8019	0.8265	0.7539	0.7912
CRPGCN	0.7965	0.7874	0.7978	0.8137	0.7954

The bold result indicates the best result in each column

**Table 2:** Comparison with other methods base on 10-CV

Method	F1-score	Accuracy	Recall	Specificity	Precision
MNCLCDA	**0.8494**	**0.8492**	**0.8469**	**0.8519**	**0.8553**
GATECDA	0.8267	0.8271	0.8312	0.8135	0.8225
MNGACDA	0.8473	0.8427	0.8436	0.8323	0.8517
VGAELDA	0.8256	0.8173	0.8332	0.8027	0.8176
MKGCN	0.8047	0.8026	0.8173	0.7973	0.7937
LAGCN	0.8133	0.8076	0.8312	0.7486	0.7956
CRPGCN	0.7977	0.7963	0.8021	0.8122	0.7927

The bold result indicates the best result in each column

26 $$\mathrm{TPR}=\frac{\mathrm{TP}}{\mathrm{TP}+\mathrm{FN}},\mathrm{FPR}=\frac{\mathrm{FP}}{\mathrm{TN}+\mathrm{FP}}$$

27 $$\mathrm{Precison}=\frac{\mathrm{TP}}{\mathrm{TP}+\mathrm{FP}},\mathrm{Recall}=\frac{\mathrm{TP}}{\mathrm{TP}+\mathrm{FN}}$$

28 $$\text{Specificity }=\frac{\text{TN}}{{\text{TN}}+{\text{FP}}}$$

29 $$F1-\text{ Score }=2\times \frac{\text{ Precision }\cdot \text{ Recall }}{\text{ Precision }+\text{ Recall}}$$

30 $$\text{Accuracy }=\frac{{\text{TP}}+{\text{TN}}}{{\text{TP}}+{\text{TN}}+{\text{FP}}+{\text{FN}}}$$

## Parameter settings

Since some parameters in the model influence its predictive performance, we use 5-CV to evaluate the main model parameters. These main parameters include the following: (1) the restart probability *c* in the RWR method, (2) the order *n* of the mixed neighbourhood during graph convolution, (3) the Gaussian kernel bandwidth γ of the kernel matrices, and (4) the Laplacian regularization coefficients ${\lambda }_{\mathrm{c}}$ and ${\lambda }_{\mathrm{d}}$. We perform experiments using a benchmark dataset and analyse the prediction performance achieved under the fivefold cross-validation setting.

**Fig. 4:**
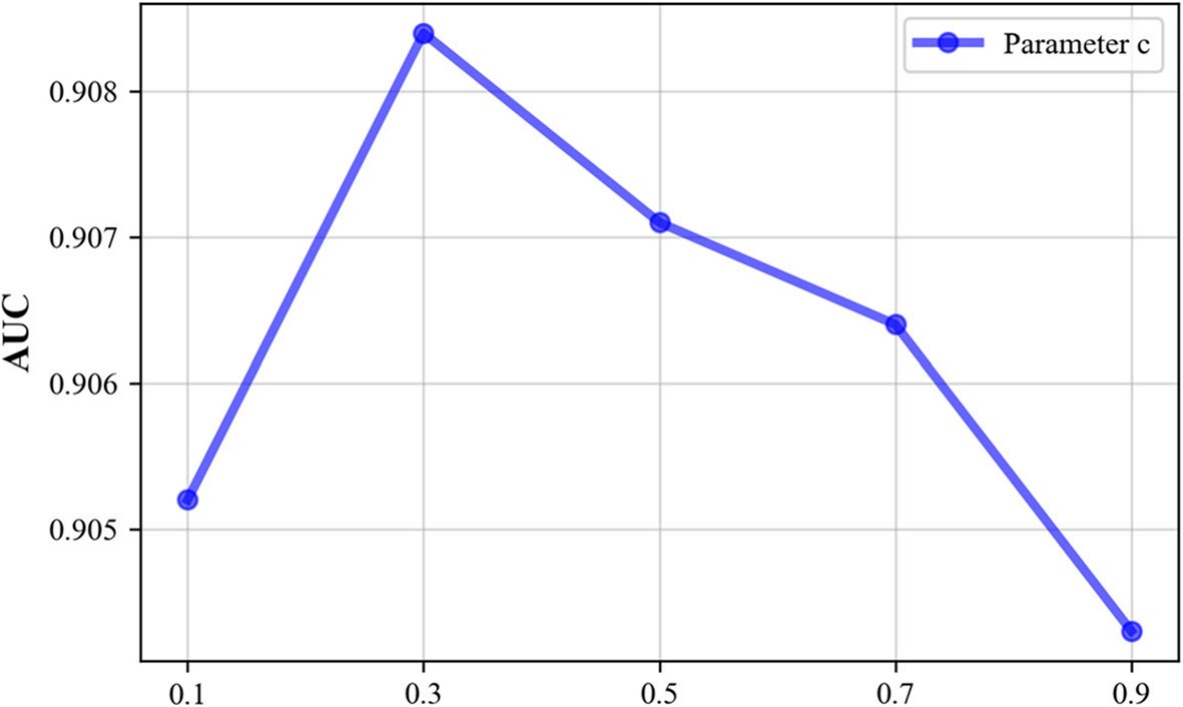
Effect of parameter *c* on the AUC

The restart probability *c* in the RWR method impacts the effectiveness of the feature extraction process, and the value of this parameter ranges from (0, 1). Therefore, we set *c* ∈ {0.1, 0.3, 0.5, 0.7, 0.9}. As shown in Fig. 4, when the restart probability *c* = 0.3, the AUC value is maximized.

**Fig. 5:**
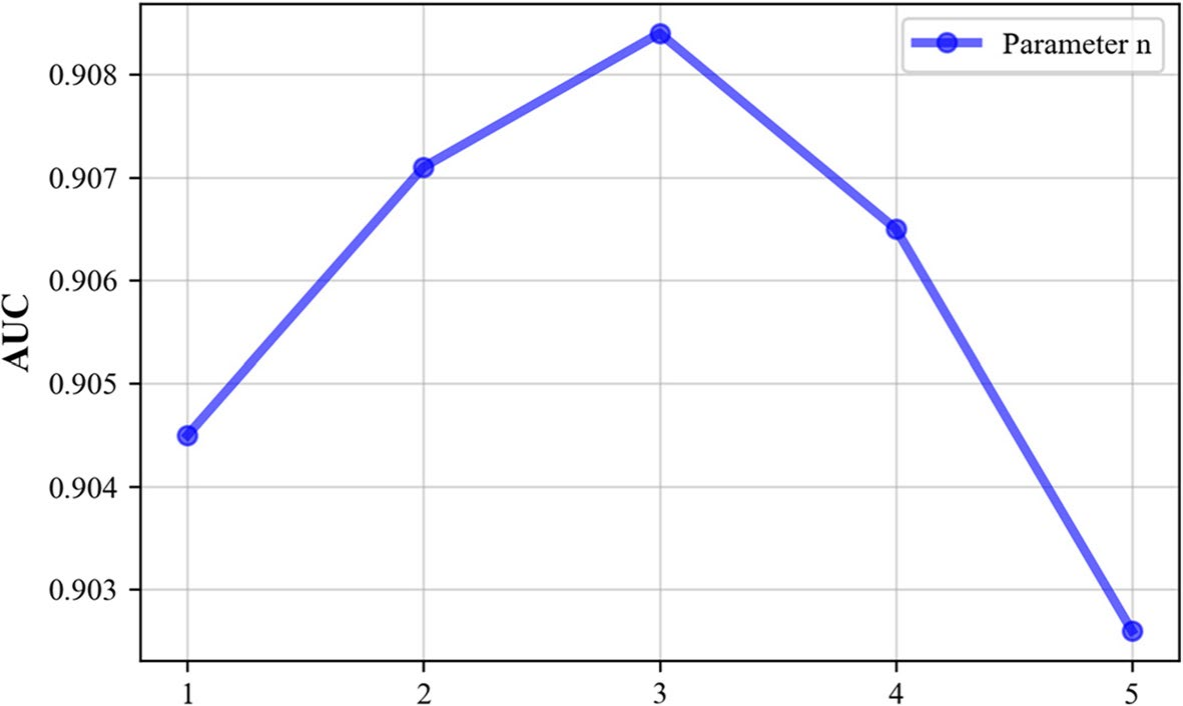
Effect of parameter *n* on the AUC

In graph convolution, the order *n* of a mixed neighbourhood indicates the farthest distance at which it can receive mixed information from its neighbours. As shown in Fig. 5, the model performance reaches the optimal state when *n* = 3.

**Fig. 6:**
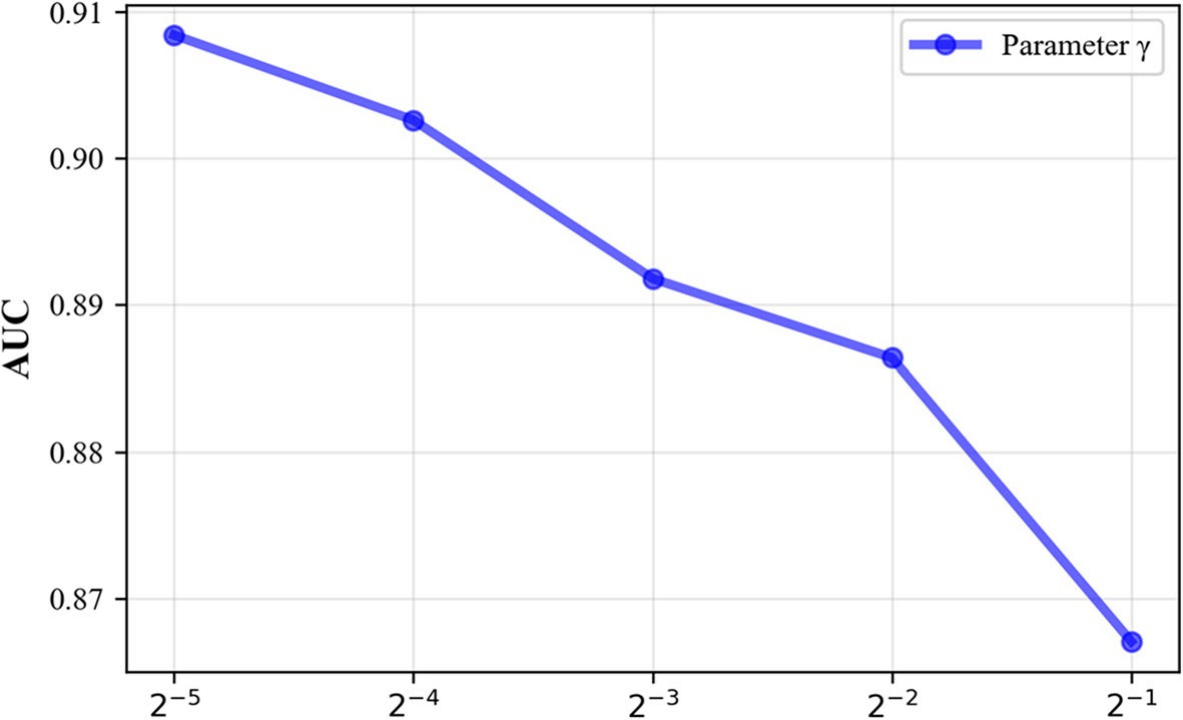
Effect of parameter γ on the AUC

The Gaussian kernel bandwidth of γ has a significant impact on the resulting prediction performance. For γ, we set the value range as γ ∈ {${2}^{-5},$ ${2}^{-4}$, ${2}^{-3}$, ${2}^{-2}$, ${2}^{-1}$}. As shown in Fig. 6, when the Gaussian kernel bandwidth γ = ${2}^{-5}$, the model performance is best.

**Fig. 7:**
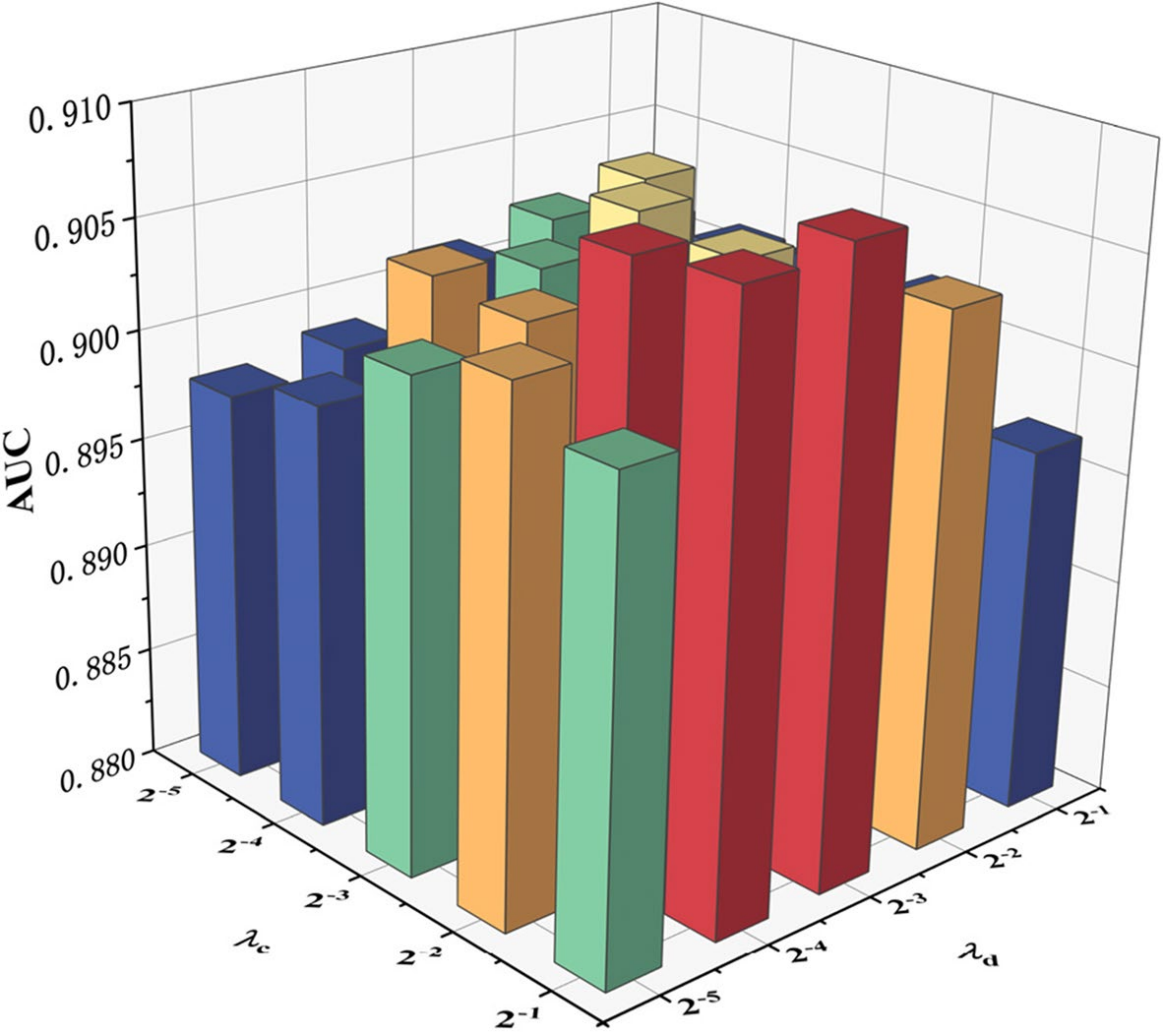
Effect of parameter ${\lambda }_{\mathrm{c}}$ and ${\lambda }_{\mathrm{d}}$ on the AUC

${\lambda }_{\mathrm{c}}$ and ${\lambda }_{\mathrm{d}}$ denote the weights of the graph regularization items in the double Laplacian-regularized least-squares method, which are important parameters. As shown in Fig. 7, when ${\lambda }_{\mathrm{c}}={2}^{-2},{2}^{-1}$, and ${\lambda }_{\mathrm{d}}={2}^{-3}$, the model has better predictive power. Therefore, our model performs best when ${\lambda }_{\mathrm{c}}={2}^{-1}$ and ${\lambda }_{\mathrm{d}}={2}^{-3}$ under the 5-CV setting.

In addition, we use Xavier [44] to initialize the parameters of the model and use the Adam optimizer [43] when training the MNCLCDA approach. The learning rate and weight decay are set to 0.005 and $1{0}^{-5}$, respectively.

## Comparison with other methods

To the best of our knowledge, very few computational methods are available for predicting the relationships between circRNAs and drug sensitivities. Therefore, to assess the predictive performance of MNCLCDA, we compare our model with six other advanced models in the field of bioinformatics. These include GATECDA [17], MNGACDA [18], LAGCN [45], MKGCN [46], CRPGCN [47] and VGAELDA [48]. In addition, the hyperparameters used in the experiment were set according to the hyperparameters recommended in the author's paper. It should be noted that in addition to GATECDA and MNGACDA, which are used in the field of circRNA-drug sensitivity association prediction, the other four known models have also been applied to other bioinformatics association prediction areas, for example, disease-circRNA and microbe–drug associations. A brief introduction to each model is given as follows.

- GATECDA [17]: a computational model for predicting the sensitivity associations between circRNAs and drugs; it uses a graph-based attentional autoencoder to extract features and finally uses a deep neural network to predict associations.
- MNGACDA [18]: a model for predicting the sensitivity associations between circRNAs and drugs; it uses a node-level graph attention-based autoencoder to extract low-dimensional representations of drugs and circRNAs from the constructed multimodal network. The final prediction process is performed using an inner product decoder.
- LAGCN [45]: a model for excavating the associations between diseases and drugs by performing graph convolution operations on heterogeneous networks and then using an attention coefficient to obtain the weights of each layer's embeddings.
- MKGCN [46]: a method for inferring microbe-drug associations using double Laplacian-regularized least-squares predictions with multiple kernel matrices.
- CRPGCN [47]: a GCN-based model that uses the RWR method and principal component analysis to extract features for predicting circRNA-disease associations.
- VGAELDA [48]: a model that integrates variational graph autoencoders and graph autoencoders for predicting lncRNA-disease associations.

**Fig. 8:**
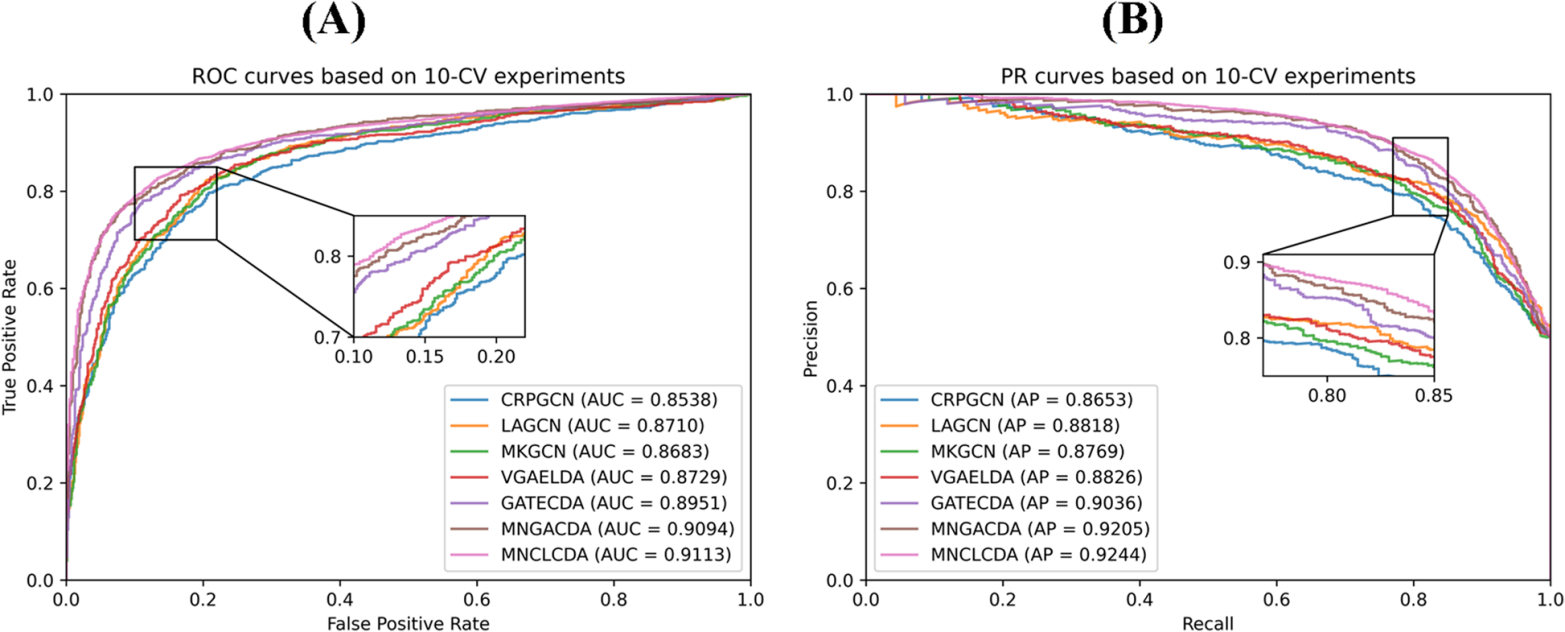
ROC and PR curves with other models on fivefold cross validation

We use the above models separately to perform cross-validation experiments on a dataset for evaluating their predictive performance. As shown in Fig. 8(A), the average AUC of MNCLCDA is 0.9084 in the 5-CV setting, which is 2.07% (GATECDA), 0.49% (MNGACDA), 4.42% (LAGCN), 4.2% (MKGCN), 5.25% (CRPGCN), and 3.66% (VGAELDA) higher than those of the competing methods. The AUPR results are shown in Fig. 8(B). The mean AUPR score of MNCLCDA is 0.9224, which is 3.14% (GATECDA), 0.78% (MNGACDA), 4.86% (LAGCN), 5.62% (MKGCN), 4.93% (CRPGCN), and 4.05% (VGAELDA) higher than those of the other methods. Furthermore, the other predictive performance indicators are shown in Table 1.

**Fig. 9:**
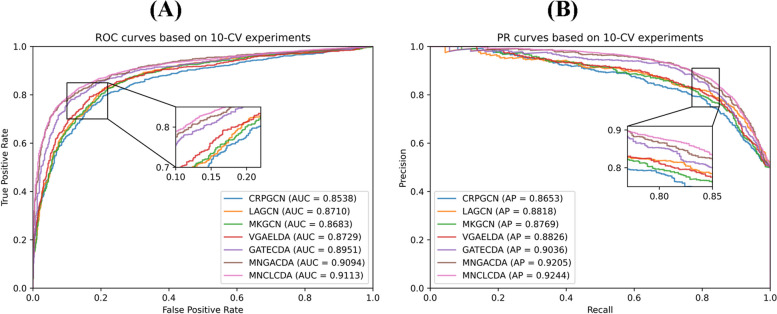
ROC and PR curves with other models on tenfold cross validation

In the 10-CV experiment, as shown in Fig. 9(A), the mean AUC score of MNCLCDA is 0.9113, which is 1.62% (GATECDA), 0.19% (MNGACDA), 4.03% (LAGCN), 4.30% (MKGCN), 5.75% (CRPGCN) and 3.84% (VGAELDA) higher than those of the other methods. The AUPR results are shown in Fig. 9(B), and the mean AUPR score of MNCLCDA is 0.9244, which is 2.08% (GATECDA), 0.39% (MNGACDA), 4.26% (LAGCN), 4.75% (MKGCN), 5.91% (CRPGCN) and 4.18% (VGAELDA) higher than those of the other methods. The other performance metrics are shown in Table 2. Overall, the above experiments show that MNCLCDA is a valid computational model for inferring circRNA-drug sensitivity associations.

## Ablation Study

This section presents a quantitative analysis of the contribution of each module in the model. Our MNCLCDA is roughly composed of four parts, including (I) the RWR-based feature processing module, (II) the mixed-neighbourhood graph convolution module, (III) the contrastive learning module and (IV) the double Laplacian-regularized prediction module. Here, we conduct an ablation experiment using 5-CV to assess the influence of each component on the predictive power of the model. Specifically, we construct the following four model variants for ablation studies. They are MNCLCDA w/o RWR, MNCLCDA w/o MN, MNCLCDA w/o CL, and MNCLCDA w/o LR, which are described as follows.

- MNCLCDA w/o RWR: RWR is removed and features are extracted using another feature extraction method (2D) PCA [49].
- MNCLCDA w/o MN: This variant uses multiple ordinary GCN layers for encoding instead of the mixed-neighbourhood GCN.
- MNCLCDA w/o CL: This version retains the other modules but does not use the contrastive learning module.
- MNCLCDA w/o LR: This version retains the other modules and uses the inner product decoder instead of the Laplacian regularized least squares for prediction.

**Fig. 10:**
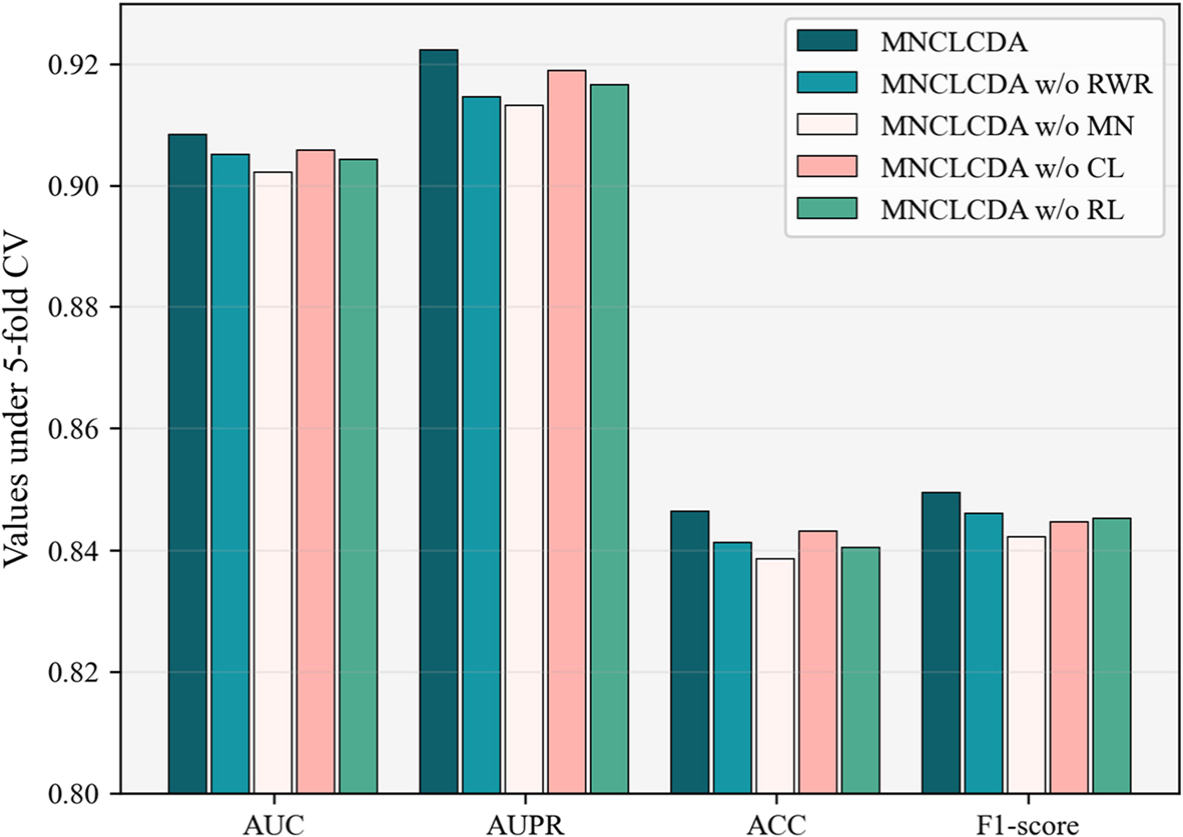
The results of MNCLCDA and its variants in the ablation study

In Fig. 10, comparisons between MNCLCDA and the four model variants in terms of performance metrics such as the AUC are shown. We note that the performance results of MNCLCDA w/o RWR show that using RWR to extract features from similar nodes can improve the predictive power of our model. Furthermore, the results of MNCLCDA w/o MN show that the embedding effect obtained by using mixed neighbourhood information is better than that obtained by using the multilayer GCN. The results of MNCLCDA w/o CL indicate that the use of the contrastive learning module also contributes to the predictive performance of the model. Finally, the results of MNCLCDA w/o LR show that the Laplace regularized least squares model is also beneficial to the predictive performance of MNCLCDA. Therefore, MNCLCDA effectively integrates the benefits of the RWR, mixed neighbourhood information, contrastive learning modules and Laplace regularized least squares model and has relatively superior performance in terms of predicting the novel associations between circRNAs and drug sensitivities.

## Case studies

The expressions of circRNA molecules impact the effects of therapeutic drugs, and they exhibit significant correlations with the effects of clinical medications [16]. To verify the effect of the MNCLCDA model in terms of predicting potential drug-related circRNA sensitivities, we conduct case studies based on the final prediction score matrix derived from the model. Specifically, we generate predictive scores by using 271 circRNAs and 218 drugs from the GDSC database as the training set. Subsequently, we randomly choose two representative cancer treatment drugs, piperlongumine and sunitinib, and rank the top 20 predicted circRNAs for the selected drugs in decreasing order based on their association scores. Since the associations between the circRNAs and drug sensitivities are obtained from the GDSC database [19], we validate the new associations predicted by the model by searching another independent database, CTRP [50].

Piperlongumine is a natural product derived from the bioactive alkaloid/amide of capsicum and capsicum longum. The pharmacological activities of piperlongumine include genotoxicity, cytotoxicity, antiangiogenic, antitumour, antiplatelet aggregation, antimetastatic, anxiolytic, antidepressant, antifungal, antibacterial, and antidiabetic activities. Among the various pharmacological effects of piperlongumine, its anticancer properties are most valuable [51, 52]. Table 3 lists the top 20 circRNAs associated with piperlongumine. After verification through the circRic (CTRP) database, 15 circRNAs have been confirmed to be associated with piperlongumine.

**Table 3:** The Top 20 circRNAs associated with the drug piperlongumine

Ranking	cricRNA	Evidence	Ranking	cricRNA	Evidence
1	PEA15 + a	CTRP	11	FBN1^a^	CTRP
2	POLR2A^a^	CTRP	12	MUC1^a^	CTRP
3	EFEMP1^a^	CTRP	13	AHNAK^a^	CTRP
4	ASPH^a^	CTRP	14	COL6A1^a^	CTRP
5	PTMS^a^	CTRP	15	MGAT4B^a^	CTRP
6	FBLN1^a^	CTRP	16	GFUS	Nonsignificant
7	COL3A1	Nonsignificant	17	ZNF609	Nonsignificant
8	MUC16^a^	CTRP	18	LINC01089^a^	CTRP
9	LTBP3^a^	CTRP	19	HSPA4	Nonsignificant
10	CTTN^a^	CTRP	20	TSPYL2	Nonsignificant

CircRNAs marked with '^a^' have been verified

Sunitinib is a small oral tyrosine kinase inhibitor molecule associated with tumour angiogenesis, and it exhibits potent antiangiogenic and antitumour activity [53, 54]. Its clinical activity was demonstrated in phase II studies involving neuroendocrine, colon, and breast cancers, while its clear efficacy was shown in advanced renal cell carcinoma and imatinib-refractory gastrointestinal mesenchymal tumours, so the FDA approved sunitinib for both diseases [54]. Table 4 lists the top 20 circRNAs associated with sunitinib. After verification through the circRic (CTRP) database, 15 circRNAs have been confirmed to be associated with sunitinib.

**Table 4:** The Top 20 circRNAs associated with the drug Sunitinib

Ranking	cricRNA	Evidence	Ranking	cricRNA	Evidence
1	KRT19^a^	CTRP	11	CRIM1^a^	CTRP
2	CTTN^a^	CTRP	12	KRT7^a^	CTRP
3	ASPH^a^	Nonsignificant	13	EVPL^a^	CTRP
4	POLR2A^a^	CTRP	14	ANXA2^a^	CTRP
5	MAL2^a^	CTRP	15	MGAT4B^a^	CTRP
6	MUC1^a^	CTRP	16	COL1A1	Nonsignificant
7	JUP^a^	CTRP	17	LTBP3^a^	CTRP
8	MUC16^a^	CTRP	18	COL1A2	Nonsignificant
9	THBS1^a^	CTRP	19	GJB3^a^	CTRP
10	ESRP2	Nonsignificant	20	COL6A2	Nonsignificant

CircRNAs marked with '^a^' have been verified

To assess the predictive performance of MNCLCDA with respect to identifying potential circRNA-drug sensitivity associations for new drugs, we select two drugs from the dataset that are associated with only one circRNA in terms of sensitivity for retesting. They are bortezomib and erlotinib. Bortezomib is the first proteasome inhibitor approved by the U.S. Food and Drug Administration (FDA) for the treatment of newly diagnosed multiple myeloma, relapsed myeloma, refractory multiple myeloma, and mantle cell lymphoma [55]. Erlotinib is a quinazoline derivative that is used to treat patients with advanced non-small-cell lung cancer (NSCLC) after the failure of platinum-containing chemotherapy [56].

In our experiments, we delete the only associations between these two drugs and their corresponding circRNAs, which are considered new drugs, while the other associations are input into the model as training set. We rank the relevant circRNAs based on the final prediction score matrix. Table 5 lists the top 10 circRNAs associated with bortezomib and erlotinib. After validation through the circRic (CTRP) database, 7 circRNAs have been validated to be associated with bortezomib, and 5 circRNAs have been validated to be associated with erlotinib.

**Table 5:** The Top 10 predicted circRNAs associated with the two new drugs: bortezomib and erlotinib

Bortezomib			Erlotinib		
**Ranking**	**cricRNA**	**Evidence**	**Ranking**	**cricRNA**	**Evidence**
1	SPINT2 + a	CTRP	1	THBS1	Nonsignificant
2	COL6A2	Nonsignificant	2	SPARC^a^	CTRP
3	DBN1^a^	CTRP	3	ASPH	Nonsignificant
4	COL1A2	Nonsignificant	4	POLR2A^a^	CTRP
5	COL1A1^a^	CTRP	5	ANXA2	Nonsignificant
6	EVPL^a^	Nonsignificant	6	PTMS	Nonsignificant
7	KRT19^a^	CTRP	7	FLNA^a^	CTRP
8	MAL2^a^	CTRP	8	FBLN1^a^	CTRP
9	COL3A1^a^	CTRP	9	COL1A1^a^	CTRP
10	MUC1	CTRP	10	EFEMP1	Nonsignificant

CircRNAs marked with '^a^' have been verified

## Discussion and conclusions

With the deepening of the research conducted on cancer and diseases, many studies have found that the expressions of circRNAs in human cells can affect the sensitivity of drugs that treat diseases, thus impacting the therapeutic effects of these drugs. Therefore, predicting the relationships between circRNAs and drug sensitivities can not only assist with the development of new drugs but also help to overcome cellular resistance to drugs, thus enhancing the therapeutic effects of these drugs for diseases. However, the use of traditional biomedical methods to identify the relationships between drugs and circRNAs is both time-consuming and laborious, so it is necessary to develop an efficient computational method for identifying potential circRNA-drug sensitivity associations, thereby reducing the cost of traditional medical experiments. In our article, we present a new computational framework, called MNCLCDA, to predict the potential associations of circRNAs with drug sensitivities. First, we construct a bipartite network based on the observed association information and then quantify the similarity between drugs and circRNAs using drug structure information, circRNA gene sequence information and Gaussian interaction features. We also use an RWR-based preprocessing mechanism to conduct feature extraction in similarity networks. Next, we obtain the embedding of the nodes by using mixed-neighbourhood graph convolution on the bipartite network. At the same time, we also design a contrastive learning task to make the model more robust. Finally, we use the double Laplacian-regularized-least squares method through the kernel matrices of the circRNA and drug spaces to infer the novel associations between the circRNAs and drug sensitivities. To verify the effectiveness of MNCLCDA, we perform cross-validation experiments on a dataset and compare our model with six related state-of-the-art computational methods. The experimental results show that our MNCLCDA model achieves the best performance. Additionally, we conduct a case study on four drugs using the proposed model and validate its results on another database, indicating that MNCLCDA is a useful tool for predicting the novel associations between drug sensitivities and circRNAs. However, the number of circRNA-drug sensitivity associates identified through biomedical experiments is still relatively small, and some bias may be present in the prediction results of the model. Collecting more circRNA-drug sensitivity associations validated by biomedical experiments can make the prediction results more reliable. In the future, we intend to collect more associations between drug sensitivities and circRNAs, as well as those involving other biological information, such as the diseases-drugs associations as well as the diseases-circRNAs associations, to enhance the predictive performance of the proposed model by using multiple sources of data. To date, the methods for predicting the associations between drug sensitivities and circRNAs are still limited, so further efforts are needed in this area.

## Data Availability

The datasets and source code are available at: https://github.com/ghli16/MNCLCDA.

## Abbreviations

circRNAs: Circular RNAs
RWR: Random walk with restart
GCN: Graph convolutional network
TPR: True positive rate
FPR: False positive rate
ROC: Receiver operating characteristic
AUC: Area under ROC curve
CV: Cross validation
